# Influence of LED light spectra on in vitro somatic embryogenesis and LC–MS analysis of chlorogenic acid and rutin in *Peucedanum japonicum* Thunb.: a medicinal herb

**DOI:** 10.1186/s40529-016-0124-z

**Published:** 2016-03-10

**Authors:** Chia-Chen Chen, Dinesh Chandra Agrawal, Maw-Rong Lee, Ren-Jye Lee, Chao-Lin Kuo, Chi-Rei Wu, Hsin-Sheng Tsay, Hung-Chi Chang

**Affiliations:** 1 Department of Chinese Pharmaceutical Sciences and Chinese Medicine Resources, China Medical University, Taichung, 40402 Taiwan; 2 Department of Golden-Ager Industry Management, Chaoyang University of Technology, Taichung, 41349 Taiwan; 3 Department of Golden-Ager Industry Management, Chaoyang University of Technology, Taichung, 41349 Taiwan; 4 Department of Chemistry, National Chung-Hsing University, Taichung, 40227 Taiwan; 5 Department of Agronomy, National Chung-Hsing University, Taichung, 40227 Taiwan

**Keywords:** Chlorogenic acid, LED lighting, *Peucedanum japonicum*, Rutin, Somatic embryogenesis

## Abstract

**Background:**

*Peucedanum japonicum* Thunb, an important medicinal herb is reported to possess pharmacological properties such as anti-obesity, anti-oxidant, anti-inflammatory, anti-bacterial, anti-diabetic and anti-platelet aggregation. The present study aimed to develop an in vitro plant regeneration system of *P. japonicum* via somatic embryogenesis and to analyse chlorogenic acid and rutin contents in a few commercially available plant products of *P. japonicum* in Japan and Taiwan markets, and tissue culture plants derived from somatic embryos.

**Results:**

Induction of somatic embryogenesis could be achieved when root derived calli after three subcultures were transferred from Murashige Skoog’s salts and vitamins (MS basal) medium with 2,4-dichlorophenoxyacetic acid (2,4-D) (0.1–5 mg/L) to a medium with abscisic acid (ABA) (0.5–4 mg/L), or exposed to eight different light spectra provided by light-emitting diode (LED) sources. Concentrations of ABA and LED light spectra had an influence on number of somatic embryos induced and proliferation of callus. Development of secondary somatic embryos and conversion of embryos to plantlets was achieved on a medium with ABA, or their exposure to red or blue lights in a special incubation chamber. Four months old tissue culture plants derived from somatic embryos showed significantly higher levels of chlorogenic acid (10.5 mg/g dw) compared to commercial product sold in Japanese market (0.55 mg/g dw). However, rutin was absent in tissue culture plants in contrast to commercial sample (0.33 mg/g dw).

**Conclusion:**

In this report, we describe in vitro plant regeneration system in *P. japonicum* via somatic embryogenesis and production of chlorogenic acid in tissue culture plants. The present study has application in further tissue culture propagation of elite plant material with high chlorogenic acid content, and identification of high yielding plants with the LC–MS method.

## Background

*Peucedanum japonicum* Thunb., (Umbelliferae), ‘the longevity herb’ is a medicinally important perennial plant. The species is distributed in Japan, the Philippines, China, Taiwan, and Korea. Leaves of *P. japonicum* have been traditionally consumed in the treatment of cough in the Yaeyama Islands (Okinawa), Japan. The roots of this herb have been used as a folk medicine for cold and neuralgic diseases in Taiwan, (Chen et al. 1996). Several coumarins isolated from roots and whole plant of *P. japonicum* have been reported to possess pharmacological activities such as anti-obesity (Nugara et al. 2014; Nukitrangsan et al. 2012; Okabe et al. 2011), anti-oxidant (Hisamoto et al. 2003), anti-inflammatory, anti-bacterial (Yang et al. 2009), anti-diabetic (Nukitrangsan et al. 2012), tyrosinase inhibition (Hisamoto et al. 2004), and anti-platelet aggregation (Chen et al. 1996). Antibacterial activities of hydro-distilled essential oils of whole parts of *P. japonicum* indicating anti-inflammatory and potential health benefits on human skin have been reported (Yang et al. 2009). Furthermore, several in vitro studies have demonstrated the occurrence of hypolipidaemic compounds in leaves of *P. japonicum* (Hsu and Yen 2007; Li et al. 2006). While studying the radical scavenging activity of *P. japonicum*, (Hisamoto et al. 2003) isolated 17 compounds from the leaves, of which several compounds played an important role in the potent antioxidant activity. Nugara et al. (2014) demonstrated that partially purified hexane phase (HP) had a crucial role in regulating lipid metabolism-related gene expression and energy expenditure in vitro, inferring that alterations in lipid metabolism rather than inhibition of lipid absorption may be the primary mechanism for the antiobesity activity of *P. japonicum*. In two other reports, the ethanol extract of aerial parts of *P. japonicum* ameliorated the symptom of diabetes in animal model by increasing the insulin sensitivity in liver, adipose, and muscle tissues (Nukitrangsan et al. 2011, 2012). Of the genes studied, *P. japonicum* could prevent the obesity via the up-regulation of FXRα that plays a pivotal role in the obesity related lipid metabolism (Nukitrangsan et al. 2011, 2012).

In somatic embryogenesis, a plant or embryo is derived from somatic cell(s) and somatic embryos are formed from plant cells that normally are not involved in the development of embryos (Bajaj 1995). Applications of somatic embryogenesis include: clonal propagation of genetically uniform plant material; elimination of viruses; provision of source tissue for genetic transformation; generation of whole plants from single cells called protoplasts; development of synthetic seeds (Bajaj 1995). Also, somatic embryogenesis has served as a model to understand the physiological and biochemical events that occur during plant development (Dudits et al. 1995). A large number of reports are available on somatic embryogenesis in a highly diverse taxonomic group of plants and studies have been periodically reviewed (Bajaj 1995; Dudits et al. 1995; Thorpe 1995; Jain and Gupta 2005; Mujib and Samaj 2006; Gutiérrez-Mora et al. 2012). The present study was carried out to develop a plant regeneration system of *P. japonicum* via somatic embryogenesis and also to carry out LC–MS analysis of chlorogenic acid and rutin contents in a few commercial products of the species marketed in Japan and Taiwan, and tissue culture plants derived from somatic embryos. Chlorogenic acid, a phenolic compound and an antioxidant has been reported to be more potent for body weight reduction and regulation of lipid metabolism than caffeic acid (Cho et al. 2010). It has been demonstrated that chlorogenic acid inhibited preadipocyte population growth, which may provide a proposed mechanism for reducing obesity (Hsu et al. 2006). Rutin, a common natural flavonoid has potential anti-tumor efficacy and anti-inflammatory effects (Deschner et al. 1991), Rutin acts as an effective inhibitor of lipid peroxidation (Yang et al. 2008) and contributes to the antibacterial (Arima et al. 2002; Watt and Pretorius 2001) and antioxidant (Ibtissem et al. 2012) properties of the plant. To the best of our knowledge, no published report has examined the effects of LED light spectra on in vitro induction of somatic embryogenesis and production of chlorogenic acid and rutin in *P. japonicum.* The present study will not only facilitate further in vitro propagation, genetic transformation of this medicinally important plant species, but also help in selecting the elite plant materials of *P. japonicum* by LC–MS analysis.

## Methods

### Plant material

Seeds of *P. japonicum* used in the present study were collected from Penghu Island in Taiwan (23°36′18.0″N 119°31′08.7″E). Commercial samples of *P. japonicum* from Japan and Taiwan were obtained from the authorized sources.

### Establishment of aseptic seedlings

Seeds of *P. japonicum* were disinfected by washing several times with sterile distilled water, followed by dipping in 70 % ethanol (v/v) for 10 s, then immersing in a solution of 0.5 % (v/v) sodium hypochlorite containing 1 drop of Tween-20 for 5 min and the step repeated three times. Final washing step carried out in a laminar flow cabinet consisted of 3 rinses of 5 min each with sterile distilled water. Thereafter, disinfected seeds were inoculated in pre-sterilized petridishes (90 mm dia). Each dish contained 20 mL of 1× Murashige and Skoog’s (1962) salts and vitamins, hereinafter referred as MS basal medium (MSBM). Gellan Gum powder (GPP) (0.4 %) purchased from PhytoTechnology Laboratories^®^ was used as a gelling agent and 3 % sucrose was supplemented to each medium. The pH of all the media was adjusted to 5.7 ± 0.1, prior addition of GPP, and before autoclaving for 15 min under 1.05 kg/cm at 121 °C. The petridishes were incubated in a culture room at 25 ± 2 °C, a light and dark cycle of 16/8 h and an illumination intensity of 34 μmol/m^2^/s. After 14 days, germinated seeds were transferred to glass bottles (650 mL capacity) each containing 100 mL of MS basal medium supplemented with 3 % sucrose and 0.4 % GPP.

### Induction of callus

For induction of somatic embryogenesis in *P. japonicum*, initial experiments were carried out to find out a suitable culture medium and an explant. Root, leaf blade and petiole parts of 35 days old in vitro raised seedlings of *P. japonicum* were used as explants. These were separately inoculated in pre-sterilized petridishes (90 mm). Each petridish contained 20 mL of MS basal medium supplemented with a range of concentrations of 2,4-dichlorophenoxyacetic acid (2,4-D) (0.1–5.0 mg/L). Each medium was supplemented and 3 % sucrose and 0.4 % GPP. Cultures were incubated in a culture room at 25 ± 2 °C in dark. After 35 days, induction of callus in each was recorded. Calli induced were subcultured for three cycles, on MSBM with same 2,4-D concentration. At the end of each subculture cycle of 35 days, fresh weight and morphological changes in the calli were recorded. Out of three explants, root showed the maximum callus proliferation, hence root callus was used for further experiments for induction of somatic embryos.

### Influence of ABA on induction of somatic embryos

To investigate the influence of ABA on induction of somatic embryos, callus was cultured in petridishes (90 mm) containing MS basal medium with abscisic acid (ABA) (0.5, 1.0, 2.0, 4.0 mg/L), 3 % sucrose and 0.4 % GPP. Five clumps of 100 mg callus was inoculated in each petridish. These were incubated in a culture room at 25 ± 2 °C in dark. After 60 days of incubation, morphological changes and induction of somatic embryos in the calli were recorded.

### Influence of different light spectra on induction of somatic embryos and callus proliferation

This experiment was performed to evaluate the influence of different light spectra on induction of somatic embryos, proliferation of callus (fresh weight) and conversion of somatic embryos into plantlets. Also, the influence of different light spectra on chlorogenic acid and rutin contents in callus was analyzed. Callus cultures were incubated in a specially designed plant growth chamber equipped with eight different LED-lights (Nano Bio Light Technology Co., Ltd., Taiwan: http://www.nanobiolight.com/en/). The chamber has two tiers of sections where pertidishes can be kept and exposed to different light spectra by specially designed LED-lids. There were total eight LED-lids (CW-5000 K, WW-2700 K, 8R1B, 7R1G1B, 3R3B3IR, 6R, 6B and 6IR). These LED-lids consisted of single or combinations of four different light spectra emitting blue (450 nm), green (525 nm), red (660 nm), and far-red (730 nm) lights. LED-lids CW-5000 K and WW-2700 K represents cool and warm white light while, 5000 K and 2700 K represents color temperature, respectively. Eight LED-lids consisted of the following ratio of light spectra in the order of blue (B): green (G): red (R): infra-red (IR); CW-5000 K (28:43:29:0), WW-2700 K (8:46:46:0), 8R1B (16:0:84:0), 7R1G1B (17:9:74:0), 3R3B3IR (57:0:43:37), 9R (0:0:100:0), 9B (100:0:0:0), 9IR (0:0:0:100) (Personal communication with the Nano Bio Light Technology Co., Ltd., Taiwan). Number (9, 7, 3, 1) in each LED-lid code represents the number of LED chips used for a particular lid. Light intensity among these eight lids varied in the range of 54–64 μmole/m2/s depending upon the type of the LED-lid. Calli were inoculated in petridishes (90 mm) which were kept in horizontal sections in the LED chamber.

### Induction of secondary somatic embryos and conversion to plantlets

Vitrified somatic embryos obtained in the ‘LED light experiment’ were cut into tiny pieces forming an embryogenic mass. This mass (200 mg/each petridish) was inoculated on a fresh MS basal medium with ABA (0.5, 1.0, 2.0, 4.0 mg/L), 3 % sucrose and 0.4 % GPP and incubated in a culture room at 25 ± 2 °C in dark. Observations were recorded after 60 days of incubation.

### Acclimation and survival of somatic embryos-derived plantlets

For further growth, small plantlets developed in the ‘LED light experiment’ were transferred to glass bottles (650 mL capacity) each containing 100 mL of MS basal medium supplemented with 3 % sucrose and 0.4 % GPP and incubated in a culture room at 25 ± 2 °C, a light and dark cycle of 16/8 h and an illumination intensity of 34 μmol/m^2^ s. When these plantlets grew about 5–6 cm in length, these were carefully taken out from the bottles, gently washed to remove adhering medium and then transplanted to plastic pots (90 mm dia) containing a mixture of peat soil: perlite: vermiculite (2:1:1). Each pot was covered with a transparent plastic sachet to maintain the humidity. These pots along with sachets were kept in the University greenhouse. After 2 weeks, sachets were removed. Survival rate was recorded after 5 weeks of transplanting.

### LC/MS analysis of tissue culture plants and commercially available samples of *P. japonicum*

#### Preparation of HPLC/MS standard and samples

Standard samples of chlorogenic acid (ChromaDex) and rutin (Fluka Analytical) were purchased from Sigma-Aldrich Co. LLC. Each standard (10 mg) was taken into a separate 10 mL volumetric flask, and then added methanol to the mark; diluted 10 times into 100 ppm with purified water; took 0.1 mL respectively and further diluted to 10 ppm. The diluted standard solution was filtered with 0.22 μm filter Millipore (Millipore, USA), and then 5 μL was injected for LC/MS analysis. Samples of calli (from the LED light spectra experiment) for the LC/MS analysis were collected from the petridishes and their fresh weights were recorded. All the samples were then freeze-dried for 24 h and their dry weights were recorded. Fraction (100 mg) of each dried sample was crushed into fine powder and dissolved in 10 mL of ethanol. It was ultra-sonicated for 10 min and the supernatant was collected after centrifugation at 5000 rpm for 10 min. This process was repeated three times for each sample. After filtration, ethanol extracts were evaporated to dryness with the help of a rotary evaporator. The residue was dissolved in 10 mL ethanol and filtered through a 0.22 μm (Millipore, USA) membrane before LC–MS analysis.

#### Instrument and the conditions

The analysis was carried out using a ‘Surveryor HPLC–MS’ pump with an auto sampler. The solution was separated on an Xbridge C18 LC column (2.1 × 150 mm, 5 µm) from Waters (Waters Corp., Milford, MA, USA) at a room temperature. The mobile phases were pure water (Millipore, USA) containing 0.1 % acetic acid (A) and acetonitrile/methanol (9/1, v/v) (B). The gradient was initialized at 80 % A held for 2 min, then increased linearly from 80 % A to 60 % A in 7 min, increased to 10 % A in the following 3 min, further increased to 5 % A in the following 5 min, and then decreased to 80 % A over 1 min. The column was then re-equilibrated at 80 % A for 4 min. The flow rate was 0.2 mL/min. Mass Spectra analyses were performed using a TSQ Quantum ultra EMR tandem Mass Spectrometer (Thermo Electron, San Jose, CA, USA), equipped with an atmospheric pressure ionization (API) interface. The spectra were obtained in positive ESI mode. The spray voltage was 4.5 kV, the capillary temperature was 275 °C, the sheath gas pressure was 35 arbitrary units, and the auxiliary gas was 5 arbitrary units. The mass scan was ranged from *m/z* 200 to 1000.

### Statistical analysis

Software SAS 9.1 was used for statistical analysis. Data were subjected to the least significant difference (LSD) tested at 5 % probability level (p > 0.05) wherever possible. Each treatment had minimum 20 replicates. The experiments were repeated three times except LC–MS analysis.

## Results and discussion

### Induction of callus and somatic embryogenesis

All three explants of *P. japonicum*, leaf blade, petiole and root induced callus at all the concentrations of 2,4-D tested (0.5–5.0 mg/L), though the quantity and quality of callus varied depending upon explant and concentration of 2,4-D (data not shown). However, there was no induction of callus on the medium devoid of 2,4-D. Induction of somatic embryos could not be observed even after 3 subcultures on 2,4-D medium. Therefore, further experiments were carried out with a range of ABA concentrations and different LED light spectra for induction of somatic embryogenesis. Since callus derived from root explants proliferated easily, hence was used for further experiments. ABA concentration in the culture medium had an influence on callus fresh weight and induction of somatic embryos. The maximum percentage of callus clumps (30 %) induced somatic embryos on medium with ABA at 4.0 mg/L, however, it induced a fewer number of somatic embryos (13) of globular stage, and the least callus proliferation per clump (172 mg). In contrast to it, a medium with 1.0 mg/L of ABA induced the maximum (44) globular stage somatic embryos, and an average 329 mg callus per clump (Table 1).

**Table 1:** Influence of abscisic acid (ABA) on induction of somatic embryos in callus of *P. japonicum*

ABA (mg/L)	Percentage of callus clumps showing somatic embryo	Total number of somatic embryos	Average callus fresh weight/clump (mg)^a^
0	15	10 (t)	447 ± 48 a
0.5	15	27 (g)	336 ± 16 b
1.0	20	44 (g)	329 ± 42 b
2.0	25	17 (g)	212 ± 27 c
4.0	30	13 (g)	172 ± 12 d

Culture medium: MS basal medium + 3 % sucrose + 0.4 % GPP. Total number of callus clumps = 20 (10 in each petridish); each callus clump = 100 mg; Observations at 60 days of culture

*g* globular, *t* torpedo

^a^Mean ± standard error. Means within each column followed by the same letter(s) are not significantly different at 5 % level by Fisher's protected LSD test

Induction of somatic embryogenesis (SE) occurs either directly when embryos develop from explant tissue without any intervening callus phase, or indirectly when an explant first produces callus which later differentiate into somatic embryos as observed in the present study. Innumerable reports have been published demonstrating both types of somatic embryogenesis in diverse taxonomic groups of plants (Bajaj 1995; Thorpe 1995; Jain and Gupta 2005; Mujib and Samaj 2006; Gutiérrez-Mora et al. 2012; Lema-Rumińska et al. 2013). Somatic embryogenesis is the developmental process by which somatic cells in a plant develop into bipolar structures without vascular connection to the parental tissue, akin to zygotic embryos. Plant growth regulators (PGRs), especially auxins play very important role in the induction of somatic embryogenesis in plants. Among different auxins, 2,4-dichlorophenoxyacetic acid (2,4-D) is one of the most common plant growth regulator applied for induction of somatic embryogenesis. 2,4-D, a synthetic and auxinic herbicide acts not only as an exogenous auxin analogue, but also as an effective stressor (Gaj 2004). In our study, both ABA and light spectra experiments, control calli also showed somatic embryos indicating that medium with 2,4-D initiated the necessary physiological changes in the calli during subcultures, however, withdrawal of 2,4-D from the medium was necessary for the development of somatic embryos. It has been observed earlier that in some cases withdrawal of auxins from the culture medium promotes induction of somatic embryogenesis in calli (Gutiérrez-Mora et al. 2012; Lema-Rumińska et al. 2013). As observed in our study, the process of induction of somatic embryos occurs through an orderly series of characteristic embryological stages like globular, heart and torpedo.

### Influence of LED light spectra on somatic embryogenesis

**Fig. 1:**
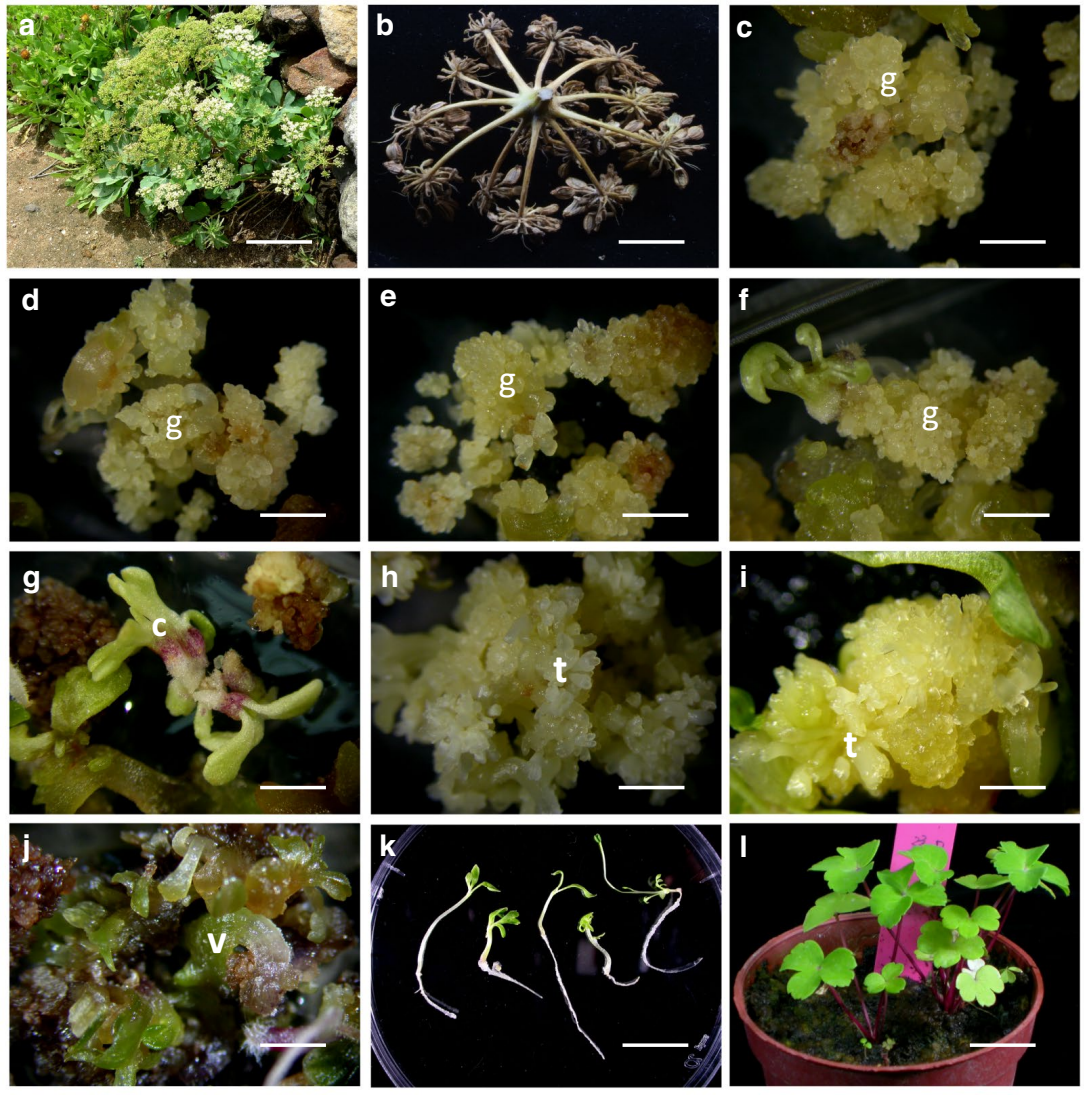
Somatic embryogenesis in *P. japonicum.* **a** Wild plants; **b** seeds of *P. japonicum;* **c**–**j** callus with somatic embryos; **k** plantlets; **l** potted plants in greenhouse. *g* globular; *c* cotyledonary; *t* torpedo; *v* vitrified embryos. *Bar*: **a** 22 cm; **b**–**j** 0.5 cm; **k** 1.4 cm; **l** 1.8 cm

Results on the influence of eight LED light spectra on development of somatic embryos, vitrification, callus proliferation (fresh weight) and callus texture is given in the Table 2. The maximum number of somatic embryos (329) in globular and torpedo stages was recorded with the light spectra 8R1B, however, the maximum callus proliferation (fresh weight) (821 mg/callus clump) was obtained with the 9IR treatment. The maximum percentage of vitrified somatic embryos (31) were recorded with the light spectrum WW. The callus texture from compact to friable, and dry to succulent varied depending upon the light spectrum it was exposed (Fig. 1c–j) Newly proliferated calli under light spectra CW and 9B showed light pink and purple color, respectively, while callus under 9IR developed deep golden color. Four light spectra 3R3B3IR, 9IR, 9R and 9B did not induce any somatic embryos (Table 2). Light quantity, quality and duration are known to control morphogenesis, growth and differentiation of plant cells, tissues and organ cultures (Moshe and Dalia 2007). Generally, fluorescent lamps, metal halide lamps, high-pressure sodium lamps or incandescent lamps are used as a source of light in tissue culture. Some of these light sources emit non-essential wavelengths of low quality (Kim et al. 2004) affecting plant growth and development. More advance light-emitting diode (LED) light sources have several advantages. LED light systems are much smaller in size, are more durable, have wavelength specificity and provide emitting surfaces relatively cool. Also, one can determine their spectral composition. LED light sources enable wavelengths to be matched to plant photoreceptors to provide more optimal production and to influence plant morphology and metabolic composition (Bourget 2008; Massa et al. 2008; Morrow 2008). Due to these advantages, LED light sources have already been used for in vitro cultivation of several commercially important plants such as upland cotton, banana, strawberry, grape, potato, maize, birch, Cymbidium, Lilium, chrysanthemum and Phalaenopsis (Saebo et al. 1995; Nhut et al. 2003; Li et al. 2010). The integration, quality, duration and intensity of red-, infrared-, blue- and ultraviolet-light can have a profound influence on plants by activating or deactivating physiological reactions and controlling their growth and development (Briggs et al. 2001; Briggs and Olney 2001; Clouse 2001). These studies have demonstrated that LED light is more suitable for plant growth than fluorescent lamps. Similar to our results, beneficial effects of LED light sources on induction of embryogenesis in *Oncidium* have been reported (Chung et al. 2010). Now LED light system is being increasingly used to boost horticulture industry in Taiwan and several other countries (Fang et al. 2011). More recently, studies on the influence of LED lighting on plant growth, physiology and secondary metabolism have been reviewed (Ouzounis et al. 2015; Olle and Versile 2013). However, the exact mechanism of how LED lights sources effect production of secondary metabolites at a molecular level is not yet known.

**Table 2:** Influence of different light spectra on induction of somatic embryos and callus growth in *P. japonicum*

Light spectrum	Total number of somatic embryos	Percentage of vitrified somatic embryos	Average callus fresh weight (mg/clump)^a^	Callus texture
CW-5000 K	110 (g)	20	732 ± 37 b	Compact, dry, pink color
WW-2700 K	90 (g + t)	31	644 ± 14 de	Compact, succulent
7R1G1B	191 (g + t)	20	635 ± 18 def	Compact, succulent
8R1B	329 (g + t)	20	555 ± 14 ef	Compact, light golden color
3R3B3IR	0	0	721 ± 16 bc	Compact, dry, with white cells
9IR	0	0	821 ± 15 a	Compact, deep golden color
9R	0	0	631 ± 6 def	Compact, light creamish color, succulent
9B	0	0	681 ± 11 bcd	New callus friable, purple color, succulent

Culture medium: MS basal medium + 3 % sucrose + 0.4 % GPP. Total number of callus clumps each petridish = 20; each callus clump = 100 mg; observations at 60 days of culture

*g* globular, *t* torpedo

^a^Mean ± standard error. Means within each column followed by the same letter(s) are not significantly different at 5 % level by Fisher's protected LSD test

### Secondary somatic embryogenesis

Secondary somatic embryogenesis (SSE) is the phenomenon whereby new embryos are initiated from somatic embryos. Thus, an unlimited number of secondary somatic embryos can be generated in a cyclic manner from a culture of primary embryos (Raemakers et al. 1995). In our study, the embryogenic mass of vitrified (hyperhydric) somatic embryos obtained in the LED light experiment when cultured on fresh MS basal medium supplemented with ABA (0.5–4 mg/L), or incubated under eight different LED-lids induced a varying number of secondary somatic embryos and developed plantlets (Fig. 1k) (data not shown). In literature, there are several contradictory reports on role of ABA in the induction of secondary somatic embryogenesis in caraway embryogenic cell clumps (Ammirato 1987), celery cell cultures (Nadel et al. 1990) and carrot cell cultures (Iida et al. 1992) indicating an interplay of several factors in affecting the secondary somatic embryogenesis.

### Survival of tissue culture plants

Conversion of somatic embryos to plantlets was observed in light spectra 9R and 9B (Fig. 1k). Plantlets could successfully be established after their transfer to plastic pots containing soil mix (peat soil:perlite:vermiculite (2:1:1) with a survival rate of 73 % in the University greenhouse.

### LC–MS analysis

Results of LC–MS analysis of chlorogenic acid and rutin contents in the commercial samples, tissue culture plants and calli from ‘LED light spectra’ experiment are presented in Table 3. The maximum chlorogenic acid content (10.5 mg/g dw) was recorded in tissue culture plants derived from somatic embryos. However, rutin could not be detected in tissue culture plants and calli obtained in ‘LED light spectra’ experiment. Among the three sources, leaf powder samples of Taiwan showed the maximum chlorogenic acid content (0.60 mg/g dw) and rutin (1.01 mg/g dw), respectively. While leaf powder samples of two different Japanese companies showed more or less similar contents of Chlorogenic acid (0.55 and 0.48 mg/g dw, respectively) and rutin (0.33 and 0.29 mg/g dw, respectively). Petiole powder sample from Taiwan also contained chlorogenic acid (0.27 mg/g dw) and rutin (0.31 mg/g dw), however root powder sample of Taiwan did not have chlorogenic acid, but showed negligible amount of rutin (0.01 mg/g dw). Calli proliferated in ‘LED light spectra’ experiment contained only chlorogenic acid in varying quantities but no rutin (Table 3). The maximum chlorogenic acid (3.44 mg/g dw) was recorded in callus exposed to light spectra ‘3R3B3IR’. Callus cultured in light spectrum ‘9R’ did not contain any of the two compounds.

**Table 3:** LC-MS analysis of chlorogenic acid and rutin in different plant materials of *P. japonicum*

Plant material	Secondary metabolites	
	Chlorogenic acid (mg/g dw)	Rutin (mg/g dw)
Leaf powder-1^a^ from Japan	0.55	0.33
Leaf powder-2^a^ from Japan	0.48	0.29
Leaf powder from Taiwan	0.60	1.01
Petiole powder from Taiwan	0.27	0.31
Root powder from Taiwan	0	0.01
Tissue culture plants (4 months old)	10.5	0
Callus—CW	0.44	0
Callus—WW	0.07	0
Callus—7R1G1B	0.20	0
Callus—8R1B	0.08	0
Callus—3R3B3IR	3.44	0
Callus—9IR	0.19	0
Callus—9R	0	0
Callus—9B	0.09	0

^a^Two leaf powders were purchased from two different companies in Japan

Similar to the present study, production of higher contents of secondary metabolite compounds by tissue culture plants compared to wild plants and commercial samples available in market has been reported earlier in medicinal plants such as *Glossogyne tenuifolia* (Chen et al. 2014), *Saussurea involucrata* (Kuo et al. 2015).

## Conclusions

In vitro induction of somatic embryogenesis and plant regeneration system in *P. japonicum* has been developed. Concentrations of ABA and different LED light spectra had a significant influence on somatic embryogenesis in *P. japonicum*. In contrast to samples obtained from markets in Japan and Taiwan, tissue culture plants had significantly higher amounts of chlorogenic acid. The study has application in micropropagation and selection of elite materials of *P. japonicum* by LC–MS method.

## Abbreviations

ABA: abscisic acid
2,4-D: 2,4-dichlorophenoxyacetic acid
LED: light-emitting diode (LED)
MS basal medium: Murashige and Skoog’s macro-, micro salts and vitamins
